# Proactive monitoring of drug–drug interactions between direct oral anticoagulants and small-molecule inhibitors in patients with non-small cell lung cancer

**DOI:** 10.1038/s41416-024-02744-1

**Published:** 2024-06-11

**Authors:** Judith L. Gulikers, Leila-Sophie Otten, Lizza E. L. Hendriks, Kristien Winckers, Yvonne Henskens, Jenneke Leentjens, Michel M. van den Heuvel, Rob ter Heine, Sander Croes, Berber Piet, Robin M. J. M. van Geel

**Affiliations:** 1 Department of Clinical Pharmacy & Toxicology, Maastricht University Medical Centre+, Maastricht, The Netherlands; 2 CARIM School for Cardiovascular Disease, Maastricht University, Maastricht, The Netherlands; 3 Department of Pharmacy, Radboud Institute for Health Sciences, Radboudumc, Nijmegen, The Netherlands; 4 Department of Respiratory Medicine, GROW – School for Oncology and Reproduction, Maastricht University Medical Centre+, Maastricht, The Netherlands; 5 Department of Internal Medicine, Maastricht University Medical Centre+, Maastricht, The Netherlands; 6 Central Diagnostic Laboratory Units for Haematology, Transfusion and Haemostasis, Maastricht University Medical Centre+, Maastricht, The Netherlands; 7 Department of Internal Medicine, Radboud Institute for Health Sciences, Radboudumc, Nijmegen, The Netherlands; 8 Department of Pulmonology, Radboud Institute for Health Sciences, Radboudumc, Nijmegen, The Netherlands

**Keywords:** Non-small-cell lung cancer, Targeted therapies

## Abstract

**Background:**

Small-molecule inhibitors (SMIs) have revolutionised the treatment of non-small cell lung cancer (NSCLC). However, SMI-induced drug–drug interactions (DDIs) with frequently co-administered direct oral anticoagulants (DOACs), increase thromboembolic and bleeding risks. This study investigated and proactively managed the consequences of DOAC-SMI DDIs.

**Methods:**

This prospective, observational study enrolled patients with NSCLC concomitantly using a DOAC and SMI. The primary outcome was the proportion of patients with DOAC plasma trough (C_trough_) and peak (C_peak_) concentrations outside expected ranges. Secondary outcomes included DOAC treatment modifications, incidence of bleeding and thromboembolic events and feasibility evaluation of pharmacokinetically guided DOAC dosing.

**Results:**

Thirty-three patients were analysed. Thirty-nine percent (13/33) had DOAC C_trough_ and/or C_peak_ were outside the expected ranges in 39% (13/33). In 71% (5/7) of patients with DOAC concentrations quantified before and during concurrent SMI use, DOAC C_trough_ and/or C_peak_ increased or decreased >50% upon SMI initiation. In all patients in whom treatment modifications were deemed necessary, DOAC concentrations were adjusted to within the expected ranges.

**Conclusion:**

Proactive monitoring showed that a substantial proportion of patients had DOAC concentrations outside the expected ranges. DOAC concentrations were successfully normalised after treatment modifications. These results highlight the importance of proactive monitoring of DOAC-SMI DDIs to improve treatment in patients with NSCLC.

## Background

Targeted therapy of non-small cell lung cancer (NSCLC) with small-molecule inhibitors (SMIs) has greatly improved the survival and quality of life of the patient population eligible for this treatment modality [1]. However, patients with cancer, including those with NSCLC, are at increased risk of developing venous thromboembolisms (VTEs) and thus, many have a vital indication for treatment with an anticoagulant [2, 3].

Direct oral anticoagulants (DOACs) are emerging as the preferred choice as treatment or secondary prevention of VTEs in patients with cancer [4]. This preference is attributed to their demonstrated non-inferior efficacy and safety profile, as evidenced by multiple double-blind randomised trials, when compared to vitamin K antagonists (VKAs) and low molecular weight heparins (LWMHs) [5–9]. Furthermore, the oral administration and the putative absence of necessity for frequent haemostasis monitoring make DOACs a more patient-friendly anticoagulant than VKAs or LMWHs. Consequently, the use of DOACs has been recommended by international guidelines for the treatment of VTE in patients with cancer [10].

The majority of SMIs used in NSCLC can cause clinically relevant drug–drug interactions (DDIs) with DOACs via inhibition or induction of their absorption, metabolism and excretion. When used concomitantly, these potential DDIs can result in increased or decreased DOAC exposure and subsequent increased risk of bleeding or thromboembolic events [11–13]. Labels of DOACs currently recommend avoiding of deviating DOAC concentrations by refraining from simultaneous administration of (strong) interacting co-medications such as certain SMIs. This now necessitates these patients transitioning to the less patient-friendly alternatives LMWHs or VKAs.

The theoretical impact of DDIs between SMIs and DOACs has been described frequently [14–16]. However, these recommendations rely on theoretical implications, lacking the necessary integration of clinical real-world data. Furthermore, polypharmacy and comorbidities, e.g. reduced renal function, are usually not accounted for in DDI recommendations. These factors make the clinical application of such recommendations complex. A reliable approach to managing these complexities could be monitoring of DOAC exposure. Therefore, we prospectively monitored DOAC concentrations in patients with NSCLC concurrently receiving SMI therapy. Additionally, we studied the effects of adjustments made to DOAC therapy.

## Methods

We performed a prospective, observational study in patients with NSCLC using SMIs and DOACs, executed in two academic hospitals in the Netherlands (Maastricht UMC+ [Maastricht] and Radboudumc [Nijmegen]). This study was approved by the medical ethical committee of the Maastricht UMC+ (NL78003.068.21/NCT05732350) and was conducted according to the Declaration of Helsinki. This study was funded by the “Academisch Alliantie Fonds”. All patients provided informed consent prior to their participation.

### Study population

Eligible patients were 18 years or older, were diagnosed with advanced NSCLC (Stage III not eligible for radical intent treatment or Stage IV), for which an SMI was indicated or already initiated, and were using or imminently starting DOAC treatment in regular care. Eligible DOAC-SMI combinations were selected using a previously published DDI-potency classification [14], which categorised interactions as irrelevant/safe, weak with unclear or unlikely relevance, moderate-potent or potent. Patients subject to a weak, moderate-potent or potent interaction were included. For newly available SMIs that were not included in the review, a pharmacist assessed potential DDIs. At the start of a new treatment, including changes in treatment, medication use, and the risk of DDI was thoroughly assessed as part of regular care. Patients on medication or supplements known to inhibit or induce cytochrome P450 3A4 (CYP3A4) or P-glycoprotein (P-gp) as described in DDI lists from The Royal Dutch Pharmacists Association and UpToDate [17–19] were excluded. Additionally, patients who were pregnant or lactating were excluded.

### Study design

**Fig. 1:**
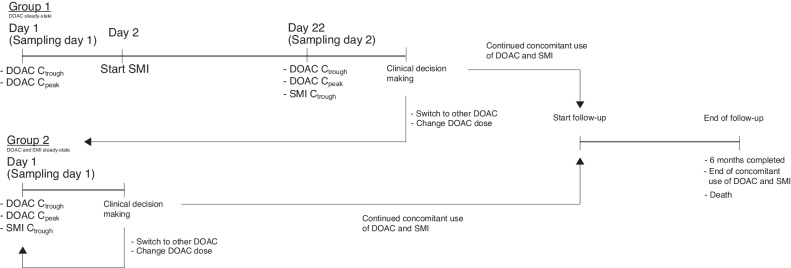
Study procedures in the two groups.

Eligible patients were enrolled in two groups (see Fig. 1). Group 1 patients already used a DOAC at maintenance dose, and an SMI was planned to start shortly after inclusion. In these patients, blood sampling to determine DOAC trough concentrations (C_trough_—sampled just before the next intake) and peak concentrations (C_peak_—sampled at ~3 h after last intake) was performed before SMI initiation (sampling day 1) and after reaching SMI and DOAC steady-state concentrations (after approximately 21 days) (sampling day 2). Group 2 patients already used a DOAC-SMI combination upon inclusion. DOAC C_trough_ and C_peak_ were determined during concomitant SMI use under steady-state conditions (sampling day 1). Quantified DOAC concentrations during concomitant SMI use were reported to the treating physicians, upon which they decided, in consultation with pharmacists and vascular physicians, whether or not to modify the DOAC treatment (i.e. adjust dose, switch to other DOAC or switch to VKA/LMWH) as part of regular care. In case of adjustments, DOAC concentrations were re-evaluated (Fig. 1). Safety follow-up started after the last sampling day and lasted up to 6 months until concomitant DOAC-SMI use stopped or death, whichever came first.

### Outcomes

The primary outcome was the proportion of patients with DOAC C_trough_ and/or C_peak_ outside the expected ranges during concurrent SMI use. Expected ranges, as defined by the Dutch Association of Medical Specialists guidelines and supplemented by data from Martin et al., were based on quantified DOAC concentrations in the general population (Supplementary Table I) [20, 21]. Secondary outcomes were: DOAC concentrations before and after concomitant SMI use, SMI C_trough_ to ensure SMI adherence, safety of the DOAC-SMI combination and the feasibility of pharmacokinetically guided dosing of DOACs in terms of adequate sampling and DOAC treatment modifications based on clinical judgement and multidisciplinary decision making. Safety assessments were conducted by the treating physician during regular outpatient visits, occurring approximately every 4 weeks to 3 months. This monitoring regimen included documentation of adverse events and blood testing, including haemoglobin. Any significant changes indicating a potential event prompted further investigation by the treating physician, with reports promptly communicated to the study team. In addition, serious adverse events (SAEs), including hospitalisation, bleeding and thromboembolic events were mandatory to register and report to the study team immediately.

### Bioanalysis

DOAC C_trough_ and C_peak_ were quantified indirectly in citrate plasma using validated clotting assays: the chromogenic anti-Xa activity DiXal (Hyphen Biomed) for aXa inhibitors and diluted thrombin time (dTT) Hemoclot (Hyphen Biomed) test for IIa inhibitor as described by Gulpen et al. [22]. SMI concentrations were quantified in EDTA plasma using LC-MS/MS methods, if available [23–25]. SMI C_trough_ was extrapolated as described by Wang et al. [26].

### Statistical analysis

Due to the cross-sectional nature of the study, no sample size calculation was performed. We aimed to include as many patients using a DOAC-SMI combination as possible between December 2021 and August 2023. Descriptive statistics were used to summarise baseline characteristics and the distribution of DOAC concentrations. Continued variables were presented as median and minimum and maximum value and categorical variables as number and percentage. The primary outcome was evaluated by calculating the proportion of patients with DOAC C_trough_ and/or C_peak_ outside the expected range. Secondary outcomes were analysed as follows; patients with DOAC C_trough_ and C_peak_ measurements before and after the addition of an SMI are described individually. Safety of DOAC treatment was evaluated by summarising bleeding and thromboembolic events during follow-up. SMI adherence was analysed by comparing the SMI C_trough_ to available SMI C_trough_ data (Supplementary Table II) [27–30]. The feasibility of pharmacokinetically guided dosing of DOACs was assessed by reporting the number of incomplete sampling days and by assessing the ability to modify DOAC treatment in patients in whom treatment modifications were deemed necessary based on clinical judgement. Analyses were performed using SAS software version 9.4 (2016) and R with Rstudio version 1.1.463 as interface and using packages “ggplot2”, “cowplot” and “forcats” [31–35].

## Results

### Patients

**Fig. 2:**
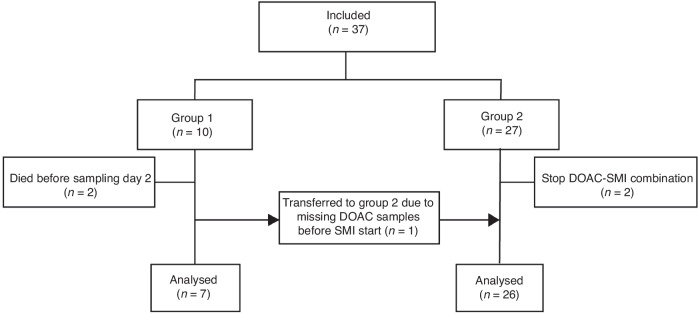
Flowchart of the study population.

Our study included 37 patients with measurements performed in 33 patients (Fig. 2). Two patients died of NSCLC, and two stopped using the SMI before sampling. The median follow-up was 4.3 months (range 0.03–6.0). Most patients used apixaban (*n* = 15) or edoxaban (*n* = 16) (Table 1). The median age was 68 years (range 34–87) and median BMI 26.0 kg/m^2^ (range 20.4– 42.2). The majority of patients (86.5%) were diagnosed with Stage IV NSCLC, with the remainder diagnosed with Stage III. DOACs were mainly prescribed for VTE treatment or prevention of its recurrence (75%). Epidermal growth factor receptor (*EGFR*), anaplastic lymphoma kinase (*ALK*) and Kirsten rat sarcoma (*KRAS*) were the most prevalent oncogenic alterations (29.7%, 18.9% and 18.9%, respectively). Accordingly, the EGFR-SMI osimertinib was used by 27.0% of all patients, the *KRAS* inhibitor sotorasib by 16.2% of the patients and the *ALK* inhibitors alectinib, crizotinib and lorlatinib by 10.8%, 8.1% and 13.5% of the patients, respectively. None of the included patients used any other medications, besides the SMI, that could have influenced DOAC pharmacokinetics or pharmacodynamics (e.g. clopidogrel or acetylsalicylic acid).

**Table 1:** Baseline characteristics.

			Total (*n* = 37)	Apixaban (*n* = 15)	Edoxaban (*n* = 16)	Rivaroxaban (*n* = 5)	Dabigatran (*n* = 1)
Sex (male), *n* (%)			19 (51.4)	8 (53.3)	9 (56.3)	1 (20)	1 (100)
Age (years), median (range)			68 (34–87)	69 (34–83)	67 (38–87)	72 (45–83)	62
BMI (kg/m^2^), median (range)			26.0 (20.0–42.4)	25.7 (20.0–31.8)	25.9 (20.4–42.4)	26.6 (24.1–33.4)	27.5
eGFR (mL/min/1.73 m^2^)^#^, median (range)			79 (44–90)	78 (40–90)	75 (58–90)	87 (48–90)	
AST (U/L), median (range)			27 (9–95)	27 (9–84)	24.5 (15–95)	27 (11–36)	NA
ALT (U/L), median (range)			23.5 (10–84)	25 (10–84)	23.5 (10–84)	22 (13–30)	NA
Bilirubin (mole/L), median (range)			6.5 (2–49.6)	7.8 (3–49.6)	6 (2–19)	4 (2–14)	NA
Albumin (g/L), median (range)			34.5 (16–42.3)	32.9 (24.1–42.3)	35 (28–38)	35 (16–39)	NA
ECOG PS, *n* (%)	0–1		31 (83.8)	12 (80)	14 (87.5)	4 (80)	1 (100)
	2		3 (8.1)	2 (13.3)		1 (20)	
	Missing		3 (8.1)	1 (6.7)	2 (12.5)		
Smoking*, *n* (%)	Never		12 (32.4)	3 (20.0)	7 (43.8)	2 (40.0)	
	Previous		22 (59.5)	10 (66.7)	8 (50.0)	3 (60.0)	1 (100)
	Current		0	0	0	0	0
	Missing		3 (8.1)	2 (13.3)	1 (6.3)		
Molecular driver, *n* (%)	EGFR		11 (29.7)	6 (40.0)	4 (25)	1 (20)	
	KRAS G12C		7 (18.9)		4 (25)	2 (40)	1 (100)
	MET exon 14 skipping		4 (10.8)	3 (20.0)	1 (6.3)		
	ROS1		5 (13.5)		5 (31.3)		
	ALK		7 (18.9)	3 (20.0)	2 (12.5)	2 (40)	
	BRAFV600E		3 (8.1)	3 (20.0)			
Metastatic disease, *n* (%)			32 (86.5)	12 (80)	16 (100)	3 (60)	1 (100)
Line of treatment, *n* (%)		1	13 (35.1)	8 (53.3)	4 (25)		1 (100)
		2	13 (35.1)	6 (40)	6 (37.5)	1 (20)	
		≥3	11 (29.7)	1 (6.7)	6 (37.5)	4 (80)	
Indication DOAC, *n* (%)	VTE		27 (73)	11 (73.3)	12 (75.0)	3 (60)	1 (100)
	Cardiac arrhythmias		8 (21.6)	3 (20.0)	4 (25.0)	1 (20)	
	Vena cava superior syndrome		1 (2.7)	1 (6.7)			
	Missing		1 (2.7)			1 (20)	
SMI, *n* (%)	Adagrasib 600 mg QD		1 (2.7)		1 (6.3)		
	Alectinib 600 mg BID		1 (2.7)	1 (6.7)			
	Alectinib, 450 mg BID		3 (8.1)	1 (6.7)	1 (6.3)	1 (20)	
	Capmatinib, 400 mg BID		3 (8.1)	3 (20.0)			
	Capmatinib, 200 mg BID		1 (2.7)		1 (6.3)		
	Crizotinib, 250 mg BID		3 (8.1)		3 (18.8)		
	Lorlatinib, 100 mg QD		5 (13.5)	1 (6.7)	3 (18.8)	1 (20)	
	Osimertinib, 80 mg QD		10 (27.0)	6 (40.0)	4 (25.0)		
	Dabrafenib, 150 mg BID		1 (2.7)	1 (6.7)			
	Dabrafenib, 75 mg BID		2 (5.4)	2 (13.3)			
	Sotorasib, 960 mg, QD		6 (16.2)		3 (18.8)	2 (40)	1 (100)
	Poziotinib, 8 mg, BID		1 (2.7)			1 (20)	
Patients in Group 1, *n* (%)			10 (27.0)	3 (20.0)	4 (25.0)	2 (40)	1 (100)

*n* number, *BMI* body mass index, *eGFR* estimated glomerular filtration rate, *AST* aspartate transaminase, *ALT* alanine transaminase, *PS* performance status, *EGFR* epidermal growth factor receptor, *KRAS* Kirsten rat sarcoma, *MET* mesenchymal epithelial transition factor receptor,* ROS1* c-ROS oncogene 1, *ALK* anaplastic lymphoma kinase, *BRAF* proto-oncogene B-RAF, *DOAC* direct oral anticoagulant, *VTE* venous thromboembolism, *SMI* small-molecule inhibitor, *QD* once daily, *BID* twice daily.

^#^Based on CKD-EPI formula [42]

*As defined by the treating physician.

### DOAC trough and peak concentrations during concurrent SMI use

In 33 unique patients, 68 DOAC C_trough_ and C_peak_ were quantified during initial DOAC-SMI use or after switching to another full-dose DOAC.

**Fig. 3:**
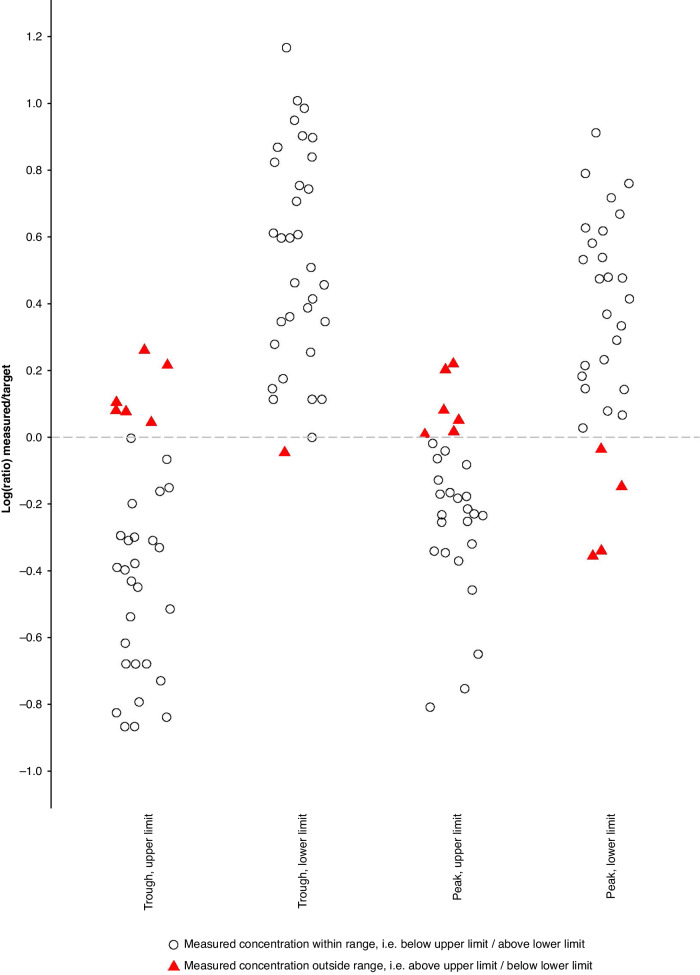
DOAC C_trough_ and C_peak_ during concomitant use of an SMI. DOAC C_trough_ and C_peak_ displayed as the ratio between the lower and upper limit of the expected range for C_trough_ and C_peak_ and the quantified DOAC C_trough_ and C_peak_. Red triangles, concentrations outside the expected range as defined in Supplementary Table I; hollow circles, concentrations within the expected ranges as defined in Supplementary Table I.

Among these patients, 13 (39.4%) had DOAC C_trough_ and/or C_peak_ outside the expected ranges (Fig. 3). For edoxaban, 6 of the 14 (42.9%) patients had the C_trough_ and/or C_peak_ outside the expected ranges, for apixaban this was the case in 4 of the 14 (28.6%) patients, for rivaroxaban in 3 of the 4 (75%) and dabigatran 0 out of 1 (0%).

Of the patients with DOAC concentrations outside the expected range, in four patients both C_trough_ and C_peak_ were deviant, in six patients only C_peak_ was deviant, and in three only C_trough_ was deviant. Individual DOAC concentrations measured during concomitant use with an SMI can be found in Supplementary Fig. I. Available SMI C_trough_ results did not show signals of non-adherence (Supplementary Table III).

### Individual DOAC concentrations before and after concomitant use of an SMI (Group 1)

**Fig. 4:**
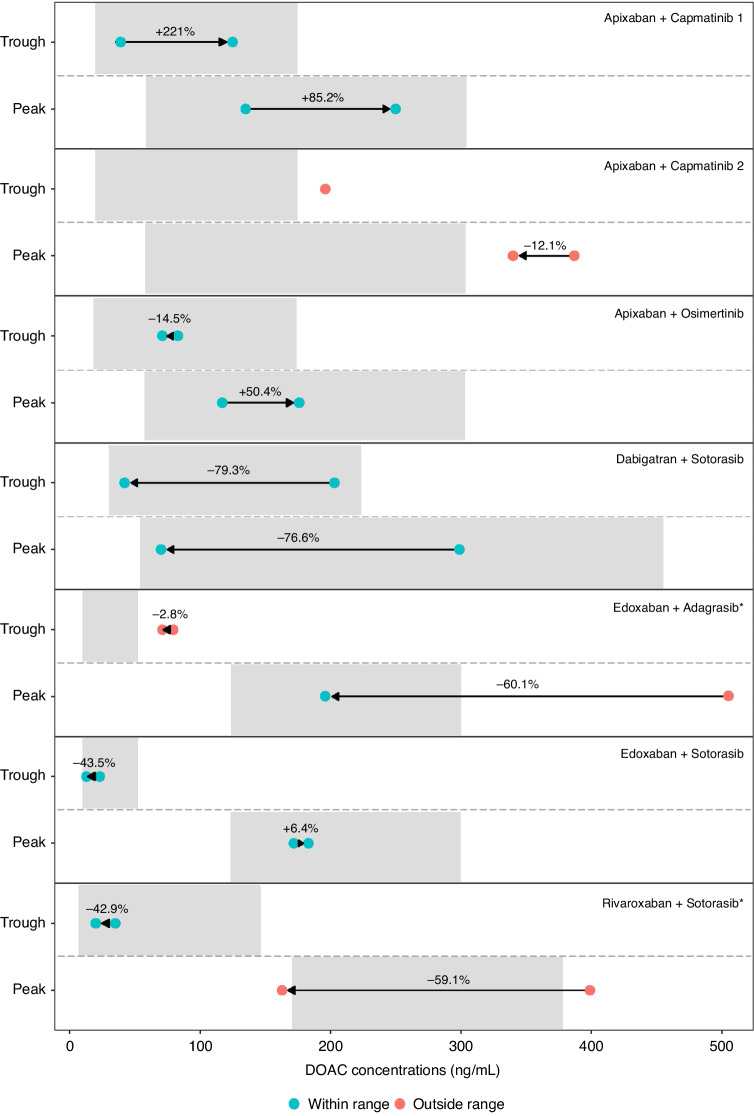
DOAC C_trough_ and C_peak_ before and after the start of concomitant SMI use in 7 patients. Grey areas indicate expected range per DOAC, red and green dots represent concentrations outside and inside the expected range, respectively, and arrows indicate the DOAC concentration change upon SMI use. *Edoxaban and rivaroxaban were reduced to 30 mg QD and 15 mg QD, respectively, between sampling day 1 and sampling day 2.

Ten patients were enrolled in Group 1 for DOAC concentration analysis before and after SMI initiation. Seven of these patients completed both sampling days (Fig. 4). Apixaban C_trough_ and C_peak_ increased by 85% and 220%, respectively, upon starting capmatinib in one patient, but led to no relevant differences in apixaban C_peak_ (13.8% decrease) in another patient. Osimertinib increased the apixaban C_peak_ by 50% without a relevant difference in C_trough_ (a decrease of 14.5%). Edoxaban with adagrasib did not result in a relevant decrease in edoxaban C_trough_ (2.8%) and C_peak_ decreased with 60.1%. Of note, edoxaban treatment was reduced to 30 mg QD between the first (i.e. prior to adagrasib use) and second sampling day (i.e. after adagrasib initiation). Edoxaban with sotorasib did not lead to relevant differences in either edoxaban C_trough_ or C_peak._. However, rivaroxaban C_trough_ and C_peak_ decreased 42.8% and 59.1%, respectively, and dabigatran C_trough_ and C_peak_ decreased 79.3% and 76.6%, respectively, upon sotorasib initiation. In the latter patient, dabigatran was switched to edoxaban 60 mg once daily (QD) due to the considerable decrease in dabigatran exposure, after which edoxaban C_trough_ and C_peak_ were within expected ranges (Supplementary Table IV). Notably, in the patient receiving rivaroxaban, rivaroxaban dose was reduced from 20 mg QD to 15 mg QD between the first and second sampling day.

### Feasibility of pharmacokinetically guided dosing

In total, 53 sampling days were performed, on which 51 and 48 adequately obtained samples were taken for DOAC C_trough_ and C_peak_ analyses, respectively. Samples without time of sampling or without time of last DOAC intake were excluded from the analysis. Whenever possible, patients were asked to revisit the hospital in case of missed sampling moments or inadequately obtained samples. The majority of missed or inadequately obtained samples were due to inappropriate timing of blood sampling or not following the strict DOAC drug intake instructions, e.g. no drug intake before the C_trough_ sampling.

**Fig. 5:**
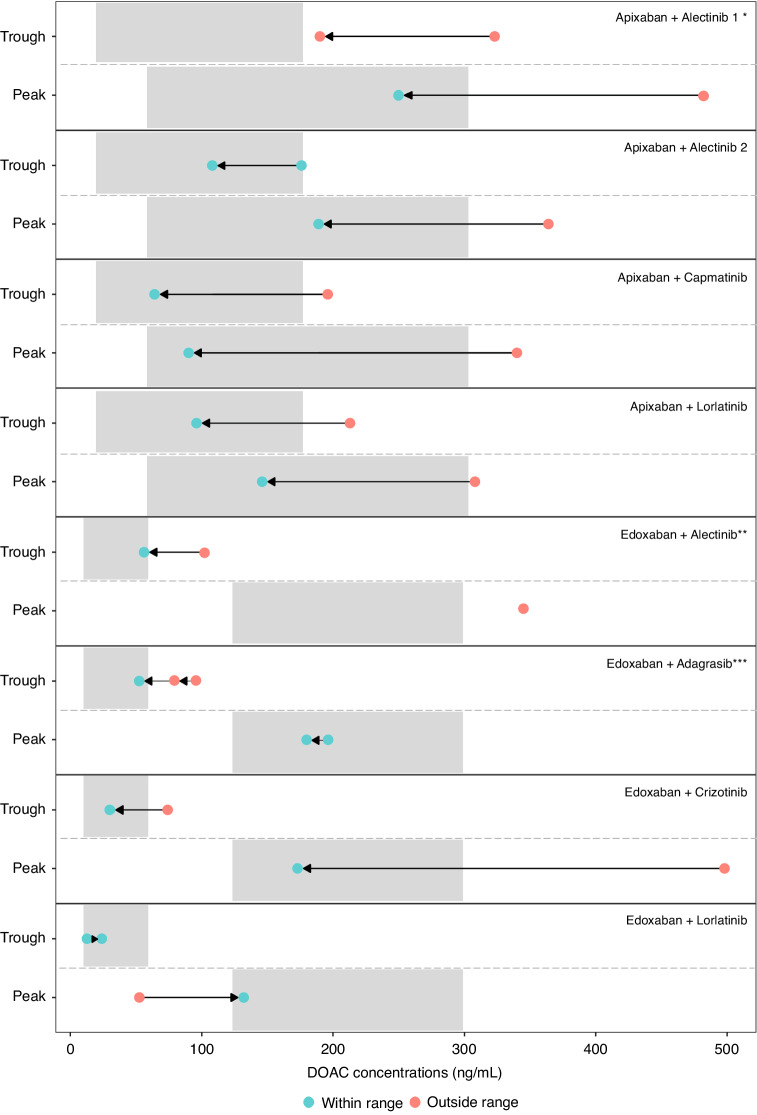
Individual DOAC C_trough_ and C_peak_ before and after DOAC dose adjustment during concomitant SMI use in 8 patients. Grey areas indicate the expected range per DOAC, red and green dots represent concentration outside and within the expected range, respectively. The arrows indicate the DOAC concentration after dose adjustment. *After dose adjustment, the apixaban C_trough_ concentration was still outside the expected range. Therefore, DOAC treatment was switched to edoxaban 60 mg QD after which edoxaban C_trough_ and C_peak_ were within the expected range. **DOAC C_peak_ measurement was missing on day 1. ***DOAC C_peak_ measurements on sampling day 2 are missing. Therefore, DOAC C_peak_ measurements are only available from sampling day 1 and day 3.

Thirteen patients had deviant DOAC concentrations, and in eight of these patients the treating physician decided to adjust the DOAC treatment. Five of these eight patients had both C_trough_ and C_peak_ above the expected range (Fig. 5). The DOAC dose was reduced in seven patients and increased in one. After DOAC dose modifications, two patients still had deviant DOAC concentrations. DOAC dose was further reduced in one patient, and one patient switched to another DOAC. After these additional modifications, all patients in whom DOAC treatment modifications were deemed necessary based on clinical judgement had DOAC concentrations within the expected ranges. No patients required a switch to a VKA or LMWH, as all DOAC concentrations eventually were within the expected ranges upon DOAC dose alteration or switching to another DOAC.

### Safety

During follow-up, no bleeding events were reported. However, one possibly related serious adverse event (SAE) occurred. In this event, the patient developed cerebrovascular ischaemia, resulting in unilateral paralysis. Notably, this patient had undergone an apixaban dose reduction due to elevated apixaban C_trough_ (213 ng/mL) and C_peak_ (308 ng/mL) at 5 mg BID. After apixaban dose reduction and before the occurrence of the SAE, all apixaban concentrations were within the expected range (C_trough_ 96 ng/mL and C_peak_ 146 ng/mL).

## Discussion

We proactively monitored DOAC pharmacokinetics in patients with NSCLC using potentially interacting DOAC-SMI combinations and found that in approximately 40%, DOAC C_trough_ and/or C_peak_ were outside the expected ranges. This strategy could facilitate achieving normalised and thereby potentially safer DOAC exposure even in the presence of DDIs.

The potential of clinically relevant DDIs between SMIs and DOACs included in the current study are previously described in a number of reviews [14–16, 36]. Although our study was not meant, nor powered, to confirm or reject the recommendations in these reviews, a number of findings are noteworthy. The most commonly used DOAC-SMI combination evaluated (apixaban and osimertinib), did so far not demonstrate apixaban concentrations outside the expected range among a limited number of evaluated patients, even though a moderate-potent interaction was expected. On the other hand, a similar potent interaction was described for alectinib in combination with apixaban or edoxaban and in our observations, dose modifications were necessary to normalise the DOAC concentration. These findings encourage to perform more DOAC-SMI DDI evaluation studies and underline the utility of proactive DOAC monitoring in case of a suspected relevant DDI, as it may also prevent unnecessary switching to another anticoagulant, such as an LMWH.

Although the number of patients with DOAC concentrations outside the expected range did not change upon addition of an SMI (i.e., in Group 1), 71.4% (5/7) of the included patients in this analysis had a DOAC C_trough_ and/or C_peak_ decrease or increase of more than 50% after SMI initiation. Given the low reported intra-patient variability in DOAC concentrations of 18–39% in a group of DOAC users without concomitant SMI use, this finding emphasizes the clinical relevance of DDIs between DOACs and SMIs [37, 38]. Especially in patients with DOAC concentrations already close to the limits of the expected range, starting concomitant use of an SMI could lead to aberrant DOAC concentrations, resulting in an elevated risk of thromboembolic or bleeding events [12, 13]. This underlines the necessity of proactive monitoring of these patients.

Our study has some limitations. We consider our study a proof-of-concept study, as our study demonstrated the effectiveness and feasibility of proactive monitoring of DOAC concentrations. Although our findings indicate that proactive DOAC treatment monitoring in patients with a suspected relevant interaction has the potential to improve care of the vulnerable NSCLC population, the primary pharmacokinetic endpoints of our study were only surrogates for clinical endpoints like thromboembolic or bleeding events. In addition, the values used to determine whether a DOAC concentration was outside the expected range are based on DOAC concentrations measured in controlled study environments, which may not be generalisable for patients with cancer. Furthermore, it may be debated whether the chosen pharmacokinetic endpoints C_trough_ and C_peak_ are the best surrogates for DOAC efficacy. Although the relationship between systemic DOAC exposure and efficacy/toxicity has been well-established, as it stands, it is currently unknown which pharmacokinetic parameter is the optimal one to guide dosing [39, 40]. On the other hand, the label recommended DOAC dosage adjustments for renal insufficiency or dose development in paediatric populations are based solely on pharmacokinetic modelling and simulation [41]. This shows that both license holders and regulatory agencies support pharmacokinetic endpoints to guide DOAC dosing. Furthermore, the sample size of our study was too small to draw definite conclusions on specific DDIs for the whole NSCLC population. We argue that the wide arsenal of DOACs and SMIs currently available and with many SMIs on the horizon, often for niche indications, it will be nearly impossible to quantify the “true” DDI potential between DOACs and SMIs for each scenario. Therefore, proactive treatment monitoring of patients with a suspected interaction appears to be a viable option to enable safe combination, but requires prospective evaluation. In addition, even though the sample size can be considered small, we argue to have included an accurate representation of patients who receive a potentially interacting DOAC-SMI combination in daily practice, considering the very limited exclusion criteria applied. Lastly, considering the real-world character of the study, it is possible to have missed noncompliance of patients to their treatment. However, none of the measured plasma concentrations do imply that there was any clinically relevant lack of treatment adherence.

To conclude, we show that suspected DDIs between SMIs and DOACs in patients with NSCLC are frequently relevant. The negative impact of such an interaction might be ameliorated using a proactive monitoring approach, but this requires prospective evaluation on clinical endpoints. Our results encourage a larger scale prospective study, using both pharmacokinetic and clinical safety and efficacy endpoints to support implementation of such a strategy in practice.

### Supplementary information

Supplementary data

## Data Availability

Data are available upon request.

## References

[1] Yuan M, Huang L-L, Chen J-H, Wu J, Xu Q. The emerging treatment landscape of targeted therapy in non-small-cell lung cancer. Signal Transduct Target Ther. 2019;4:61. 31871778 10.1038/s41392-019-0099-9 PMC6914774

[2] Mulder FI, Horváth-Puhó E, van Es N, van Laarhoven HWM, Pedersen L, Moik F, et al. Venous thromboembolism in cancer patients: a population-based cohort study. Blood. 2021;137:1959–69. 33171494 10.1182/blood.2020007338

[3] Mahajan A, Brunson A, Adesina O, Keegan THM, Wun T. The incidence of cancer-associated thrombosis is increasing over time. Blood Adv. 2022;6:307–20. 34649273 10.1182/bloodadvances.2021005590 PMC8753193

[4] Riaz IB, Fuentes H, Deng Y, Naqvi SAA, Yao X, Sangaralingham LR, et al. Comparative effectiveness of anticoagulants in patients with cancer-associated thrombosis. JAMA Netw Open. 2023;6:e2325283–e. 37486628 10.1001/jamanetworkopen.2023.25283 PMC10366701

[5] Schrag D, Uno H, Rosovsky R, Rutherford C, Sanfilippo K, Villano JL, et al. Direct oral anticoagulants vs low-molecular-weight heparin and recurrent VTE in patients with cancer: a randomized clinical trial. JAMA. 2023;329:1924–33. 37266947 10.1001/jama.2023.7843 PMC10265290

[6] Raskob GE, van Es N, Verhamme P, Carrier M, Di Nisio M, Garcia D, et al. Edoxaban for the treatment of cancer-associated venous thromboembolism. New Engl J Med. 2018;378:615–24. 29231094 10.1056/NEJMoa1711948

[7] Agnelli G, Becattini C, Meyer G, Muñoz A, Huisman MV, Connors JM, et al. Apixaban for the treatment of venous thromboembolism associated with cancer. New Engl J Med. 2020;382:1599–607. 32223112 10.1056/NEJMoa1915103

[8] McBane RD 2nd, Wysokinski WE, Le-Rademacher JG, Zemla T, Ashrani A, Tafur A, et al. Apixaban and dalteparin in active malignancy-associated venous thromboembolism: The ADAM VTE trial. J Thromb Haemost. 2020;18:411–21. 31630479 10.1111/jth.14662

[9] Young AM, Marshall A, Thirlwall J, Chapman O, Lokare A, Hill C, et al. Comparison of an oral factor Xa inhibitor with low molecular weight heparin in patients with cancer with venous thromboembolism: results of a randomized trial (SELECT-D). J Clin Oncol. 2018;36:2017–23. 29746227 10.1200/JCO.2018.78.8034

[10] Farge D, Frere C, Connors JM, Khorana AA, Kakkar A, Ay C, et al. 2022 international clinical practice guidelines for the treatment and prophylaxis of venous thromboembolism in patients with cancer, including patients with COVID-19. Lancet Oncol. 2022;23:e334–e47. 35772465 10.1016/S1470-2045(22)00160-7 PMC9236567

[11] Rizos T, Meid AD, Huppertz A, Dumschat C, Purrucker J, Foerster KI, et al. Low exposure to direct oral anticoagulants is associated with ischemic stroke and its severity. J Stroke. 2022;24:88–97. 35135063 10.5853/jos.2020.04952 PMC8829480

[12] Reilly PA, Lehr T, Haertter S, Connolly SJ, Yusuf S, Eikelboom JW, et al. The effect of dabigatran plasma concentrations and patient characteristics on the frequency of ischemic stroke and major bleeding in atrial fibrillation patients: the RE-LY trial (randomized evaluation of long-term anticoagulation therapy). J Am Coll Cardiol. 2014;63:321–8. 24076487 10.1016/j.jacc.2013.07.104

[13] Ruff CT, Giugliano RP, Braunwald E, Morrow DA, Murphy SA, Kuder JF, et al. Association between edoxaban dose, concentration, anti-Factor Xa activity, and outcomes: an analysis of data from the randomised, double-blind ENGAGE AF-TIMI 48 trial. Lancet. 2015;385:2288–95. 25769361 10.1016/S0140-6736(14)61943-7

[14] Otten LS, Piet B, van den Heuvel MM, Marzolini C, van Geel R, Gulikers JL, et al. Practical recommendations to combine small-molecule inhibitors and direct oral anticoagulants in patients with nonsmall cell lung cancer. Eur Respir Rev. 2022;31. 10.1183/16000617.0004-2022 PMC9489148 35705208

[15] Van der Linden L, Vanassche T, Van Cutsem E, Van Aelst L, Verhamme P. Pharmacokinetic drug–drug interactions with direct anticoagulants in the management of cancer-associated thrombosis. Br J Clin Pharmacol. 2023;89:2369–76. 37170893 10.1111/bcp.15785

[16] Hellfritzsch M, Henriksen JN, Holt MI, Grove EL. Drug–drug interactions in the treatment of cancer-associated venous thromboembolism with direct oral anticoagulants. Semin Thromb Hemost. 2023. 10.1055/s-0043-1762596 36731488

[17] UpToDate. Inhibitors and inducers of P-glycoprotein (P-gp) drug efflux pump (p-gp multidrug resistance transporter). https://www.uptodate.com/contents/image/print?imageKey=EM%2F73326&topicKey=HEME%2F1370&source=see_link.

[18] UpToDate. Cytochrome P450 3A (including 3A4) inhibitors and inducers. https://www.uptodate.com/contents/image?imageKey=CARD%2F76992.

[19] Kennisbank K Interactielijsten: KNMP. https://kennisbank.knmp.nl/article/Informatorium_Medicamentorum/G1126.html#G2055.

[20] Martin K, Beyer-Westendorf J, Davidson BL, Huisman MV, Sandset PM, Moll S. Use of the direct oral anticoagulants in obese patients: guidance from the SSC of the ISTH. J Thromb Haemost. 2016;14:1308–13. 27299806 10.1111/jth.13323 PMC4936273

[21] Specialisten FM. Antitrombotisch beleid: Federatie Medisch specialisten; 2020 [updated 24-3-2021. https://richtlijnendatabase.nl/richtlijn/antitrombotisch_beleid/periprocedureel_beleid_bij_antistolling.html.

[22] Gulpen AJW, Ten Cate H, Henskens YMC, van Oerle R, Wetzels R, Schalla S, et al. The daily practice of direct oral anticoagulant use in patients with atrial fibrillation; an observational cohort study. PLoS ONE. 2019;14:e0217302. 31170727 10.1371/journal.pone.0217302 PMC6554016

[23] van Veelen A, van Geel R, de Beer Y, Dingemans AM, Stolk L, Ter Heine R, et al. Validation of an analytical method using HPLC-MS/MS to quantify osimertinib in human plasma and supplementary stability results. Biomed Chromatogr. 2020;34:e4771. 31808583 10.1002/bmc.4771

[24] van Veelen A, van Geel R, Schoufs R, de Beer Y, Stolk LM, Hendriks LEL, et al. Development and validation of an HPLC–MS/MS method to simultaneously quantify alectinib, crizotinib, erlotinib, gefitinib and osimertinib in human plasma samples, using one assay run. Biomed Chromatogr. 2021;35:e5224. 34363425 10.1002/bmc.5224

[25] Gulikers JL, van Veelen AJ, Sinkiewicz EMJ, de Beer YM, Slikkerveer M, Stolk LML, et al. Development and validation of an HPLC-MS/MS method to simultaneously quantify brigatinib, lorlatinib, pralsetinib and selpercatinib in human K2-EDTA plasma. Biomed Chromatogr. 2023;37:e5628. 36941218 10.1002/bmc.5628

[26] Wang Y, Chia YL, Nedelman J, Schran H, Mahon FX, Molimard M. A therapeutic drug monitoring algorithm for refining the imatinib trough level obtained at different sampling times. Ther Drug Monit. 2009;31:579–84. 19730279 10.1097/FTD.0b013e3181b2c8cf

[27] van Veelen A, Veerman GDM, Verschueren MV, Gulikers JL, Steendam CMJ, Brouns A, et al. Exploring the impact of patient-specific clinical features on osimertinib effectiveness in a real-world cohort of patients with EGFR mutated non-small cell lung cancer. Int J Cancer. 2023;154:332–42. 10.1002/ijc.34742 37840304

[28] Chen J, Houk B, Pithavala YK, Ruiz-Garcia A. Population pharmacokinetic model with time-varying clearance for lorlatinib using pooled data from patients with non-small cell lung cancer and healthy participants. CPT Pharmacomet Syst Pharm. 2021;10:148–60. 10.1002/psp4.12585 PMC7894400 33449423

[29] Groenland SL, Geel DR, Janssen JM, de Vries N, Rosing H, Beijnen JH, et al. Exposure-response analyses of anaplastic lymphoma kinase inhibitors crizotinib and alectinib in non-small cell lung cancer patients. Clin Pharm Ther. 2021;109:394–402. 10.1002/cpt.1989 PMC7891593 32686074

[30] Moreno V, Greil R, Yachnin J, Majem M, Wermke M, Arkenau H-T, et al. Pharmacokinetics and safety of capmatinib with food in patients with MET-dysregulated advanced solid tumors. Clin Ther. 2021;43:1092–111. 34053700 10.1016/j.clinthera.2021.04.006

[31] Wickham H. ggplot2: elegant graphics for data analysis. Springer-Verlag New York; 2016 [Available from: https://ggplot2.tidyverse.org].

[32] Wilke CO. cowplot: streamlined plot theme and plot annotations for ‘ggplot2’; 2020 [Available from: https://wilkelab.org/cowplot]

[33] Wickham H. forcats: tools for working with categorical variables (factors); 2023 [Available from: https://forcats.tidyvers.org]

[34] Team R. RStudio: Integrated Development for R.: RStudio, Inc., Boston, MA; 2016 [Available from: http://www.rstudio.com/].

[35] Cary N SAS version 9.4. USA: Inc. SI.; 2016.

[36] Bolek H, Ürün Y. Cancer-associated thrombosis and drug-drug interactions of antithrombotic and antineoplastic agents. Cancer. 2023;129:3216–29. 10.1002/cncr.34937 37401828

[37] Toorop MMA, van Rein N, Nierman MC, Vermaas HW, Huisman MV, van der Meer FJM, et al. Inter- and intra-individual concentrations of direct oral anticoagulants: the KIDOAC study. J Thromb Haemost. 2022;20:92–103. 34664401 10.1111/jth.15563 PMC9297950

[38] Testa S, Tripodi A, Legnani C, Pengo V, Abbate R, Dellanoce C, et al. Plasma levels of direct oral anticoagulants in real life patients with atrial fibrillation: results observed in four anticoagulation clinics. Thromb Res. 2016;137:178–83. 26672898 10.1016/j.thromres.2015.12.001

[39] Bernier M, Lancrerot SL, Parassol N, Lavrut T, Viotti J, Rocher F, et al. Therapeutic drug monitoring of direct oral anticoagulants may increase their benefit-risk ratio. J Cardiovasc Pharmacol. 2020;76:472–7. 33030858 10.1097/FJC.0000000000000870

[40] Jelliffe R, Christians U. The new direct-acting oral anticoagulants need to be monitored! Ther Drug Monit. 2020;42:357–9. 32427780 10.1097/FTD.0000000000000745

[41] Röshammar D, Huang F, Albisetti M, Bomgaars L, Chalmers E, Luciani M, et al. Pharmacokinetic modeling and simulation support for age- and weight-adjusted dosing of dabigatran etexilate in children with venous thromboembolism. J Thromb Haemost. 2021;19:1259–70. 33636042 10.1111/jth.15277 PMC8251571

[42] Levey AS, et al. (2009) A new equation to estimate glomerular filtration rate. Ann Intern Med. 2009;150:604–12. 10.7326/0003-4819-150-9-200905050-00006 PMC2763564 19414839

