# Supplementary material for: Proactive monitoring of drug–drug interactions between direct oral anticoagulants and small-molecule inhibitors in patients with non-small cell lung cancer
Source: Br J Cancer. 2024 Jun 11;131(3):481–90. doi: 10.1038/s41416-024-02744-1 (PMC11300802; doi:10.1038/s41416-024-02744-1)
Supplement: Supplementary file 1 — Supplementary data [file 41416_2024_2744_MOESM1_ESM.docx]

**Supplementary table I.** Expected DOAC C_trough_ and C_peak_ ranges adopted from Martin *et al.* and antithrombotic policy from Dutch Association of Medical Specialists guideline (20, 21).

| **DOAC** | **Standard dose** | **Trough concentration (ng/mL) (range)** | **Peak concentration (ng/mL) (range)** |
| --- | --- | --- | --- |
| Apixaban | 5 mg BID | 22 – 177 | 59 – 302 |
| Edoxaban | 60 mg QD | 10 – 62 | 120 – 300 |
| Rivaroxaban | 20 mg QD | 9 – 147 | 177 – 361 |
| Dabigatran | 150 mg BID | 30 – 225 | 60 – 450 |
| Abbreviations; DOAC, direct oral anticoagulant; BID, twice daily; QD, once daily | | | |

**Supplementary table II.** Arbitrarily chosen minimum steady-state SMI C_trough_ values that can be expected with regular dosages for each SMI based on historical data (27-30) .

| **SMI** | **Minimum plasma C_trough_ (ng/mL)** |
| --- | --- |
| Adagrasib | Not determined |
| Alectinib | 300 |
| Capmatinib | 300 |
| Crizotinib | 120 |
| Dabrafenib | Not determined |
| Lorlatinib | 60 |
| Osimertinib | 100 |
| Sotorasib | 63 |

**Supplementary figure I.** Individual DOAC C_trough_ (blue) and C_peak_ (orange).


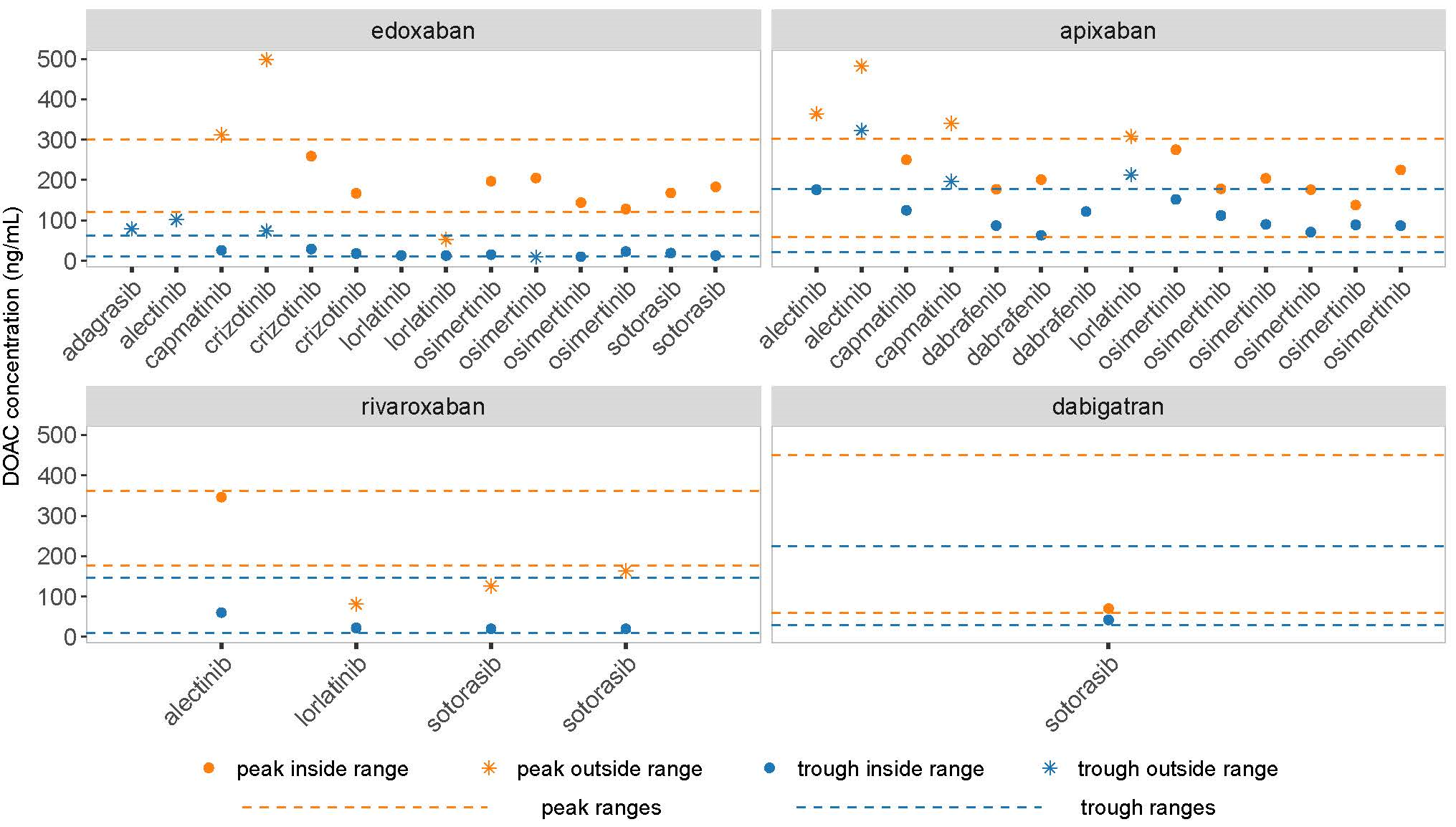


**Supplementary table III.** Observed ranges of quantified SMI C_trough_ (ng/mL) in steady-state of participants of this study with available SMI samples_._

| **SMI (n)** | **Range (ng/mL)** |
| --- | --- |
| Alectinib, 600 mg BID (1) | 366 |
| Alectinib, 450 mg BID (2) | 490 – 898 |
| Capmatinib, 400 mg BID (2) | 711 - 744 |
| Crizotinib, 250 mg BID (3) | 126 – 401 |
| Lorlatinib, 100 mg QD (4) | 90 – 191 |
| Osimertinib, 80 mg QD (10) | 130 – 648 |
| Sotorasib, 960 mg QD (4) | 126 – 294 |
| Abbreviations: BID, twice daily; QD, once daily | |

**Supplementary table IV.** DOAC C_trough_ and C_peak_ before and after concomitant use of an SMI in patients in group 1.

| DOAC | SMI | Body weight  (kg) | DOAC without SMI | | DOAC with SMI, measurement 1 | | | | DOAC with SMI, measurement 2 | | | |
| --- | --- | --- | --- | --- | --- | --- | --- | --- | --- | --- | --- | --- |
|  |  |  | DOAC | | DOAC | | SMI | Decision DOAC | DOAC | | SMI | Decision DOAC |
|  |  |  | C_trough_ (ng/mL) | C_peak_ (ng/mL) | C_trough_ (ng/mL) | C_peak_ (ng/mL) | C_trough_ (ng/mL) |  | C_trough_ (ng/mL) | C_peak_ (ng/mL) | C_trough_ (ng/mL) |  |
| Apixaban | Capmatinib | 91 | 39 | 135 | 125 🡅 | 250 🡅 |  | Continue |  |  |  |  |
|  |  | - | - | **387** | **196** | **340** |  | Reduce DOAC dose to 2.5 mg BID | 90 | 64 | - | Continue |
|  | Osimertinib | 104 | 83 | 117 | 71 | 176 🡅 | 174 | Continue |  |  |  |  |
| Edoxaban | Adagrasib* |  | **71** | **500** | **79** | - | - | Reduce DOAC to 15 mg QD | 52 | 180 | - | Continue |
|  | Sotorasib | 112 | 23 | 172 | 13🡇 | 183 | 119 | Continue |  |  |  |  |
| Rivaroxaban | Sotorasib** | 68 | 35 | **399** | 20 🡇 | 163 🡇 | - | Continue |  |  |  |  |
| Dabigatran | Sotorasib | 87 | 203 | 299 | 42 🡇 | 70 🡇 | 206 | Switch to edoxaban 60 mg QD | 25 | 209 | 167 | Continue |
| Abbreviations; DOAC, direct oral anticoagulant; SMI, small molecule inhibitor; C_trough_, trough concentration; C_peak_, peak concentration; BID, twice daily; QD, once daily; 🡅>50% increase in DOAC concentration, 🡇 >50% decrease in DOAC concentration. DOAC concentrations outside the expected range are depicted in bold. *Edoxaban dose was reduced to 30 mg QD after Sampling day 1. **Rivaroxaban dose was reduced to 15 mg QD after Sampling day 1. | | | | | | | | | | | | |
